# Hypoxia inducible factor-1ɑ as a potential therapeutic target for osteosarcoma metastasis

**DOI:** 10.3389/fphar.2024.1350187

**Published:** 2024-01-24

**Authors:** Jianghu Zhou, Fengjun Lan, Miao Liu, Fengyan Wang, Xu Ning, Hua Yang, Hong Sun

**Affiliations:** ^1^ Department of Orthopaedics, Affiliated Hospital of Guizhou Medical University, Guiyang, China; ^2^ Department of Orthopaedics, West China Hospital, Sichuan University, Chengdu, China

**Keywords:** osteosarcoma, HIF-1ɑ, metastasis, cellular microenvironment, signaling pathways, therapy

## Abstract

Osteosarcoma (OS) is a malignant tumor originating from mesenchymal tissue. Pulmonary metastasis is usually present upon initial diagnosis, and metastasis is the primary factor affecting the poor prognosis of patients with OS. Current research shows that the ability to regulate the cellular microenvironment is essential for preventing the distant metastasis of OS, and anoxic microenvironments are important features of solid tumors. During hypoxia, hypoxia-inducible factor-1α (HIF-1α) expression levels and stability increase. Increased HIF-1α promotes tumor vascular remodeling, epithelial-mesenchymal transformation (EMT), and OS cells invasiveness; this leads to distant metastasis of OS cells. HIF-1α plays an essential role in the mechanisms of OS metastasis. In order to develop precise prognostic indicators and potential therapeutic targets for OS treatment, this review examines the molecular mechanisms of HIF-1α in the distant metastasis of OS cells; the signal transduction pathways mediated by HIF-1α are also discussed.

## 1 Introduction

Osteosarcoma (OS) is the most common primary bone malignancy, derived from primitive bone-forming mesenchymal cell, for which there are two peak periods of incidence in adolescents and older adults (Shoaib et al., 2022). OS typically develops near the end of the long bone in the lower limbs, with high local invasiveness, rapid infiltration, and early metastasis (Vailas et al., 2019; Li K. et al., 2023a). The pathogenic characteristic of OS, a spindle stromal cell tumor, is the direct conversion of proliferating tumor cells into bone or tissue that resembles bone from the perspective of pathomorphology (Dong et al., 2022). Histologically, OS encompasses various subtypes including conventional, telangiectatic, small cell, high-grade surface, secondary, low-grade central, periosteal, and parosteal variants. The conventional type of OS (intramedullary high-grade) accounts for approximately 85% of all cases, making it the most prevalent subtype (Rickel et al., 2017). Neoadjuvant radiotherapy and chemotherapy combined with limb salvage surgery is the primary OS treatment method at present (Anderson, 2016). In recent years, with the improvement of chemotherapy and the introduction of immunotherapy and targeted therapy, the prognosis and overall survival of OS patients have improved (Yang G. et al., 2021a). The prognostic survival rate of OS patients with distant metastasis, nevertheless, remains significantly low (Berner et al., 2015; Bläsius et al., 2022).

Metastasis means that the cells from an original tumor location invade the surrounding tissue and colonize a new site, through the vascular system or lymphatic duct (Valastyan and Weinberg, 2011). Metastasis, tumor location, and size are the main factors affecting the poor prognosis of OS, especially metastasis (Smeland et al., 2019). A significant proportion of individuals diagnosed with OS, ranging from 20%–30%, already presented with lung metastases at the time of their initial diagnosis (Chen et al., 2021). Patients with metastasis at diagnosis or recurrence was only a 20% 5-year survival rate (Meazza and Scanagatta, 2016). Significantly, recently conducted studies have demonstrated a robust correlation between modifications in the cellular microenvironment and the occurrence of OS metastasis (Jin J. et al., 2023a).

**FIGURE 1:**
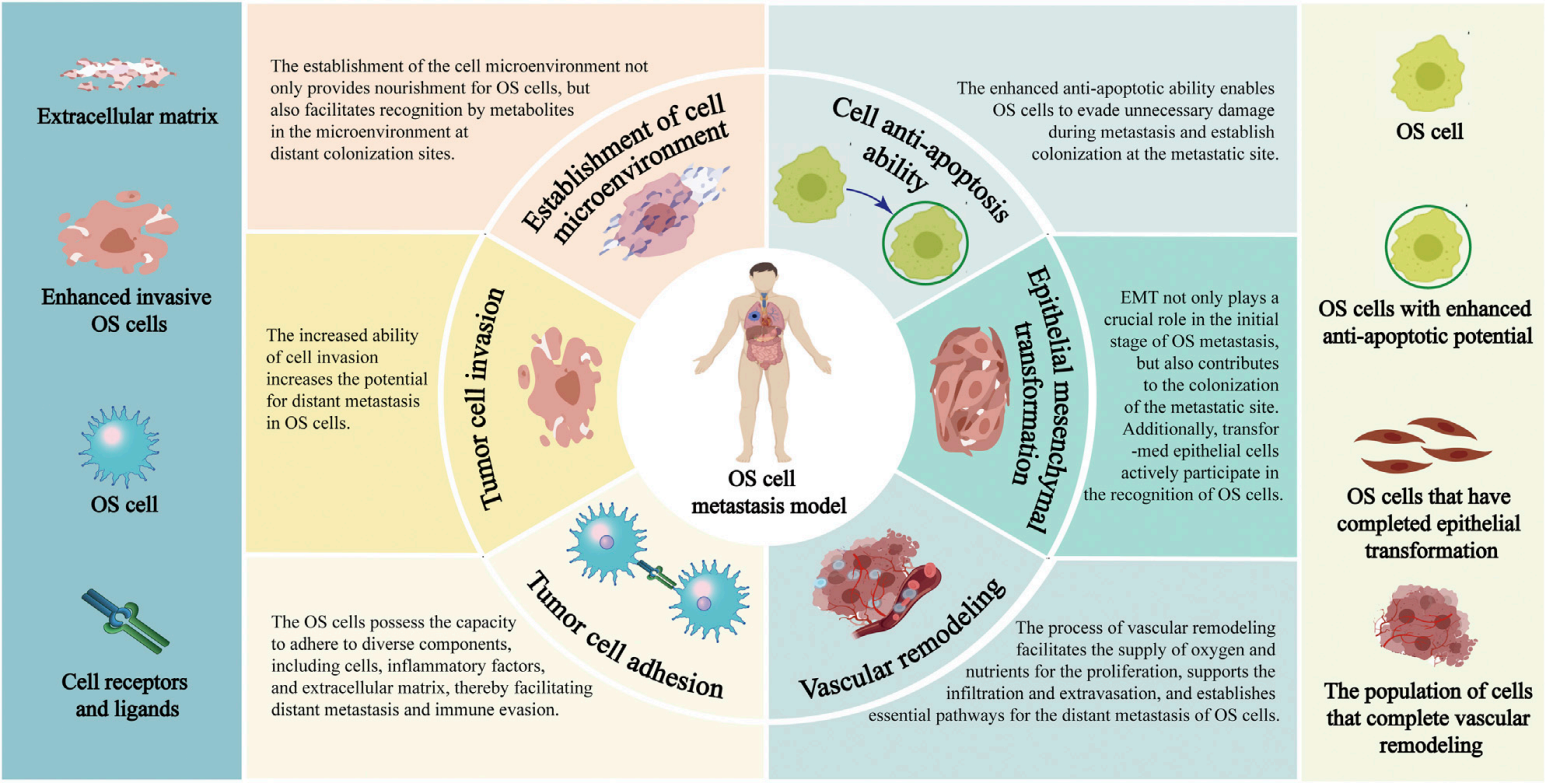
Pathological process of distant metastasis of OS. Annotation: The pathological mechanisms of distant metastasis of OS include: Enhance the invasiveness of OS cells; Facilitate the EMT process; Reduce the adhesion ability of OS cells; Enhance the anti-apoptosis characteristics of OS cells; Reconstruction of extracellular environment; Vascular remodeling process of OS cells.

The tumor cells are experiencing hypoxia as a result of their rapid cellular proliferation, heightened oxygen demand, vascular remodeling, and compromised blood supply (Li Y. et al., 2021a). The heterogeneity of hypoxia in tumor cells is characterized by varying degrees, ranging from minimal to mild to severe, and the regulatory mechanism varies depending on the degree of hypoxia (Hompland et al., 2021). Hypoxia-induced modifications in the cellular microenvironment can lead to the activation of multiple signaling pathways (Wu et al., 2023). The presence of the intricate mechanisms regulating hypoxia undoubtedly presents greater challenges in the treatment of malignancies. It is widely acknowledged that the hypoxia-inducible factor-1α (HIF-1α) is one of the most critical regulators in cellular microenvironment (Ildiz et al., 2023). The activation of HIF-1α facilitates rapid tumor cell adaptation to hypoxic environments, thereby contributing to the metastasis process of various malignant tumors (Ma et al., 2021). The regulation of the anoxic microenvironment is also associated with the mechanism of OS metastasis, enabling control over tumor cell invasion and metastasis by manipulating the hypoxic cellular microenvironment to modulate tumor cell heterogeneity (Mohamed et al., 2023) (Figure 1).

In this review, we comprehensively reviewed the molecular mechanisms of HIF-1α in the progression of OS metastasis, alongside the intricate correlation between HIF-1α and the prognosis of patients with metastatic OS as well as relevant therapeutic strategies. Deep insights into the underlying mechanisms implicated in OS metastasis would help improve the prognosis, and provide novel therapeutic targets for patients with metastatic OS.

## 2 Biological characteristics and functions of HIF-1α

The equilibrium of oxygen content in extracellular fluid is crucial for cell survival and normal metabolism (Tao et al., 2021). Under severe hypoxic stress, cells precisely regulate the expression of certain coding genes or non-coding RNA through oxygen receptors and signal transduction, thereby participating in a variety of physiological and pathological processes (Duan et al., 2024). The hypoxia-inducible factors are members of the basic helix-loop-helix Per-Arnt-Sim transcription factor superfamily. This heterodimer consists of an oxygen concentration-sensitive HIF-α subunit and a constitutively-expressed HIF-β subunit (Semenza and Wang, 1992; Wang and Semenza, 1993). HIF-α subunit includes three subtypes: HIF-1α, HIF-2α, and HIF-3α (Nangaku and Eckardt, 2007). At present, the role and function of HIF-1α have been extensively studied compared to other subtypes (Ravenna et al., 2016).

Under normoxic conditions, HIF-1α is unstable, continuously degraded, and maintained at a relatively low basal level (Li L. et al., 2023b). The prolyl hydroxylase domain (PHD) catalyzes the hydroxylation of proline residues at positions 402 and 564 on the HIF-1α subunit. Hydroxylated HIF-1α is recognized by an E3 ubiquitin ligase complex, that includes the tumor suppressor protein von Hippel-Lindau (VHL), leading to its rapid degradation via the ubiquitin-proteasome pathway. The hydroxylase activity of PHD and HIF-1α is inhibited under hypoxia. After translocation into the nucleus, HIF-1α binds to HIF-1β, forming a transcriptional complex that binds to the hypoxia response element (HRE) in the promoter region of its target gene, which initiates the transcriptional expression of many downstream genes and participates in a variety of physiological and pathological processes (Haase, 2017). In addition to being regulated by oxygen concentration, HIF-1α is also subject to regulation by various other factors, including the antisense transcription factor aHIF-1α, which has a negative regulatory effect on the transcription of the HIF-1α gene (Bertozzi et al., 2011). Certain growth factors, inflammatory factors, and oncogenes can also regulate HIF-1α protein stability through signal pathways, such as phosphoinositide 3-kinase (PI3K)/protein kinase B (PKB/AKT) and extracellular regulated protein kinases 1/2 (ERK1/2) (Zhao et al., 2024). Additionally, multiple miRNAs are also involved in regulating the expression of HIF-1α (Lee et al., 2015).

**FIGURE 2:**
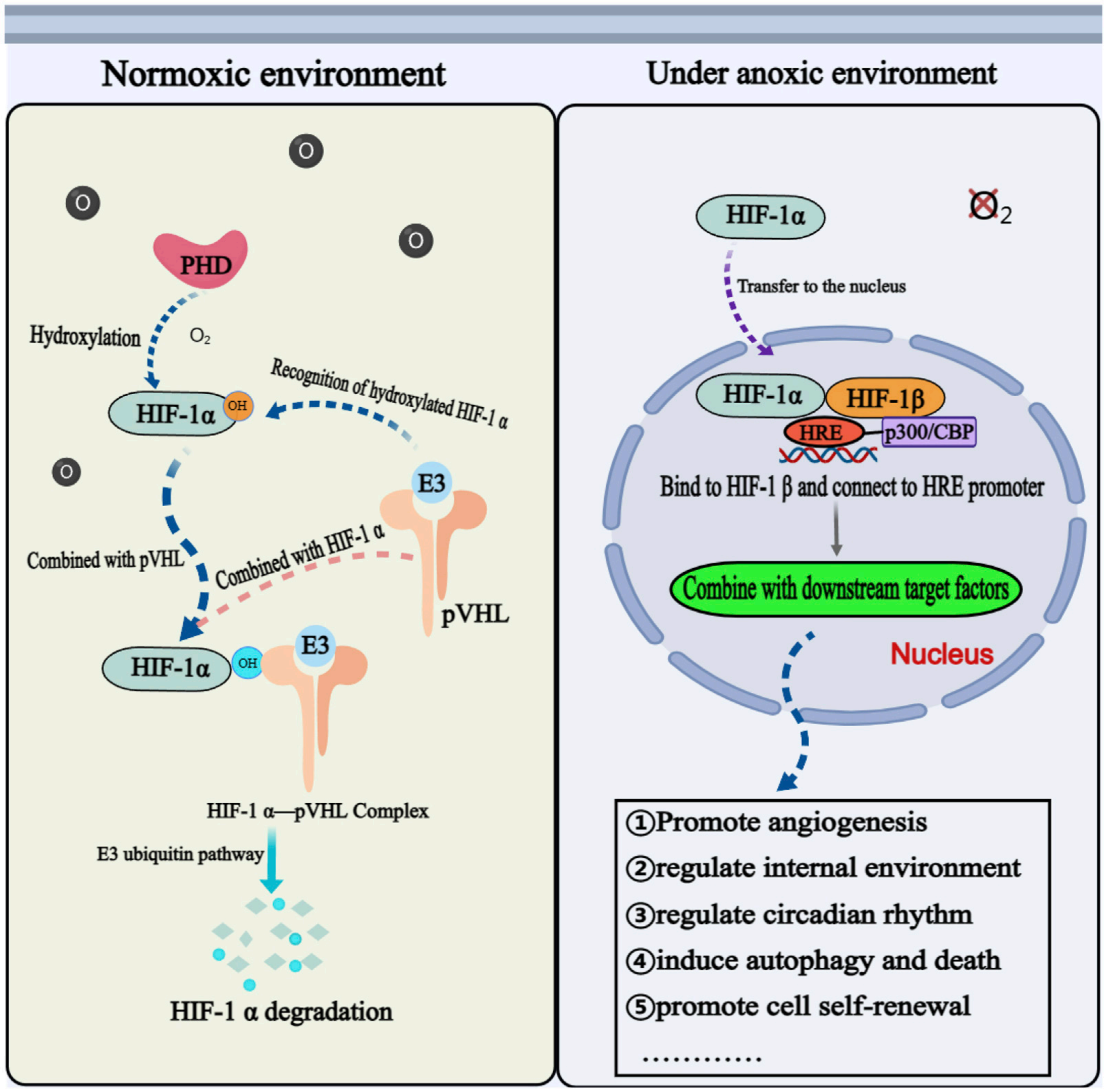
The physiological mechanism of HIF-1ɑ in normoxic and hypoxic environments. Annotation: The mechanism of HIF-1α is different under anoxic and aerobic conditions. Under aerobic condition, HIF-1α cannot exist stably and needs to be degraded through related mechanisms. Under anoxic condition, HIF-1α is activated and participates in the physiological and pathological process of cells through corresponding signal pathways.

Many studies have shown that HIF-1α is implicated in a majority of biological functions, such as promoting angiogenesis, regulating the internal environment, regulating circadian rhythm, inducing autophagy and programmed cell death, and promoting self-renewal and differentiation of mesenchymal stem cells (Adamovich et al., 2017; Wu et al., 2017) (Figure 2). The elevated expression level of HIF-1α has been observed in various primary and secondary malignant tumors, making it a valuable biomarker and potential target for clinical diagnosis, targeted treatment, and prognosis evaluation in numerous diseases (Semenza, 2012; Zeng et al., 2014; Tuomisto et al., 2016). Studies also indicated that HIF-1α plays a crucial role in regulating various molecular stages of OS metastasis and demonstrates significant prognostic implications (Khojastehnezhad et al., 2022). Therefore, HIF-1α may serve as a prognostic indicator for OS metastasis and a potential therapeutic target to enhance the prognosis and survival rate of patients with OS.

## 3 Mechanisms of malignant tumor metastasis

**FIGURE 3:**
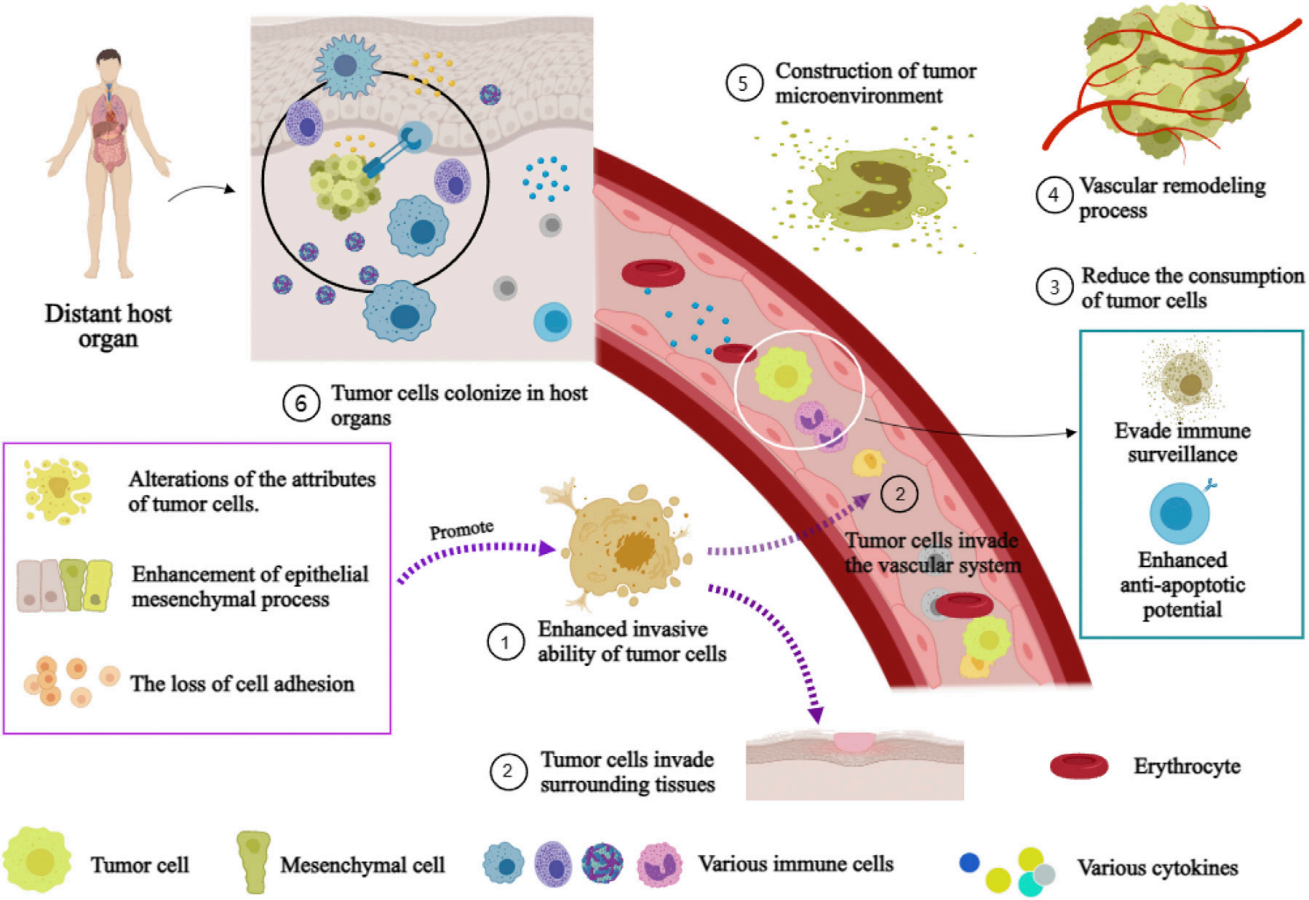
Mechanism of distant Metastasis of malignant tumor. Annotation: The mechanism of distant metastasis in malignant tumors: Firstiy, the enhancement of cell invasiveness, enabling tumor cells to invade surrounding tissues and the vascular system. Subsequently, tumor cells infiltrate the vascular system and surrounding tissues. Immune escape and anti-apoptosis mechanisms within the vasculature while reducing their loss. Finally, host organ microenvironments facilitate tumor cell colonization.

The molecular mechanism underlying the initiation and sustenance the metastasis of tumor is intricate (Tanaka and Sakamoto, 2023). The mechanism encompasses the establishment of a tumor microenvironment, the process of vascular remodeling, the acquisition of tumor cell invasiveness, and the reduction in tumor cell consumption (Figure 3). The initiation and maintenance of the metastatic process in malignant tumor cells involve the coordinated interaction and mutual influence of multiple pathways.

### 3.1 Acquisition of cell invasiveness

The acquisition of invasiveness is the primary determinant influencing distant metastasis in tumor cells. As a result of alterations in cellular characteristics, the facilitation of epithelial-mesenchymal transition (EMT), and the disruption of intercellular adhesion, there is an augmentation in the invasive capacity of tumor cells (Zepeda-Enríquez et al., 2023). After the enhancement of the invasive ability of tumor cells, they can invade the vascular system or surrounding tissue through the extracellular matrix (Huang et al., 2020). EMT is a process of transformation from polarized epithelial cells to mesenchymal cells, which drives tumor progression by enhancing the invasive ability of tumor cells (Huang et al., 2023). The process of EMT is crucial for facilitating the infiltration of OS cells into the local vascular system and promoting their migration to distant organs (Sinha et al., 2020). Additionally, the presence of established epithelial phenotypic cells further facilitates the proliferation of secondary tumors in distant organs (Hinton et al., 2023). Cell adhesion is also an important factors in tumor cell metastasis. Cell adhesion molecules plays a pivotal role in adhesion, triggers intracellular signal transduction, and participates in the regulation of EMT, thereby participating in the progression of distant metastasis of tumor cells (Ruan et al., 2022).

### 3.2 Reduced consumption of tumor cells

Evading immune system monitoring is a crucial process in minimizing tumor cell elimination and facilitating their metastasis (Elanany et al., 2023). It has been shown that disrupting the tumor vascular system and recruiting aPDL1-loaded platelets can coordinate the response of tumor cells to immune factors and control malignant tumor metastasis (Li H. et al., 2021b). The anti-apoptosis mechanism enables tumor cell survival in the circulation through the vascular system is another way to reduce the consumption of tumor cells (Zhou et al., 2023). The presence of the immune escape and anti-apoptosis mechanism can attenuate the consumption of tumor cells during metastasis, giving tumor cells a better chance to travel to and populate the metastatic location.

### 3.3 Construction of tumor microenvironment

The establishment of a tumor cell ecological environment involves the coexistence of tumor cells with other tumor cells or host cells within the same microenvironment. From a holistic perspective, the tumor cell environment can be regarded as a complex tumor ecosystem consisting of diverse biological and abiotic factors that interact with each other, and influencing the progression of tumor development through various mechanisms (Bullman, 2023; Gong et al., 2023). The initiation of metastasis is accompanied by alterations in the cellular microenvironment, which enables tumor cells to become more invasive (Chen and Song, 2022). Prior to the infiltration of metastatic tumor cells into the host organ, the coordinated actions of cytokines, exosomes, and metabolites help to create a pre-metastatic milieu in particular organs prior to the entry of metastatic tumor cells and aid circulating tumor cells in completing colonization (Peinado et al., 2017). Surprisingly, the acidification milieu and elevated extracellular lactate levels also play a vital role in the immunological regulation of tumor cells and the promotion of neovascularization (Gottfried et al., 2006; Végran et al., 2011; Brand et al., 2016; Rastogi et al., 2023).

### 3.4 Vascular remodeling effect

The neovascularization of solid tumors is considered as a pivotal process in the advancement and dissemination of tumors (Kikuchi et al., 2019). Tumor cells, tumor-associated stromal cells, and their bioactive products collectively contribute to the regulation of angiogenesis within tumor tissues (De Palma et al., 2017). On vascular remodeling, vascular endothelial growth factor (VEGF) is one of them that has a significant impact (Alsina-Sanchis et al., 2021). In tumors, the secretion of VEGF is typically continuous, leading to aberrant angiogenesis and structural disarray within the vascular system. As a consequence, tumor tissue hemoperfusion is compromised, along with intercellular junction integrity is missing. This dysfunctional vascular network further facilitates hypoxia and immunosuppression, ultimately fostering tumor progression (Khan and Kerbel, 2018). In another aspect, the remodeling of tumor vasculature plays a significant role in EMT, cell invasion, cellular infiltration, immunosuppression, and regulation of the cellular microenvironment (Tzeng and Huang, 2023). Moreover, a remodeled endothelium within capillaries can promote the metastatic colonization of tumor cells (Karreman et al., 2023).

In brief, the process of tumor cell distant metastasis is a multifaceted and intricate multistage phenomenon, encompassing numerous pivotal steps (Liu QL. et al., 2021a). Regulation involves multiple factors that interact with each other. Finding significant targets is crucial for exploring the mechanism of OS distant metastasis. The majority of the aforementioned processes are regulated by HIF-1α, which may serve as a crucial hub in controlling OS metastasis.

## 4 Molecular mechanisms of HIF-1α in OS

HIF-1α, the most significant regulator of the cellular response to anoxic environments, is associated with OS cell occurrence, development, and treatment resistance (Han and Shen, 2020). Keratin 17 is a vital component of keratin, and is capable of inducing OS cell development and glycolysis, which are indispensable for cellular energy supply by activating the AKT/mTOR/HIF-1α pathway in the underlying mechanism of OS growth (Yan et al., 2020). During the process of OS cell proliferation, mitochondrial H_2_O_2_ signal transduction promotes tumor cell proliferation through HIF-1α-dependent and HIF-1α-independent manners (Sabharwal et al., 2023). Another study revealed that HIF-1α and VEGF regulate OS cell apoptosis under hypoxia, and any disruption to one substance may subsequently affect the existence of the other, demonstrating the critical function that HIF-1α and VEGF play in OS apoptosis (Yang SY. et al., 2021b). Moreover, the mechanism of HIF-1α inducing OS cells growth was confirmed through the urothelial cancer associated 1/protein tyrosine phosphatase/AKT signal pathway (Li T. et al., 2018a).

Furthermore, there is also a strong association between the expression of HIF-1α and the mechanism of drug resistance in OS cells. Visfatin can enhance miRNA expression through HIF-1α-induced transcription. However, visfatin does not affect zinc finger e-box binding homeobox 1 mRNA expression but significantly increases its protein stability. Decreasing visfatin levels can ameliorate the sensitivity of drug-resistant OS cells to cisplatin (Wang D. et al., 2019a). HIF-1α is directly targeted by miR-199a, and there is an inverse correlation between miR-199a and HIF-1α mRNA. Overexpression of MiR-199a resensitizes cisplatin resistant OS cells to cisplatin by inhibiting HIF-1α both *in vitro* and *in vivo* (Keremu et al., 2019). The following study presented that miR-216b enhances cisplatin induced apoptosis by regulating the JMJD2C/HIF-1α/Hairy and enhancer of split 1 signal axis in OS cells, which may be a potential strategy for overcoming chemical resistance in OS (Yang et al., 2020). In another interesting study, transforming growth factor β downregulates the expression of succinate dehydrogenase by reducing the levels of transcription factor STAT1. This leads to upregulation of HIF-1α, which disrupts glucose metabolism and exacerbates chemical resistance in OS cells (Xu et al., 2021). However, the involvement of HIF-1α extends beyond its role in the occurrence and drug resistance of OS, as it also exhibits a close association with the progression and metastasis of OS (Shang et al., 2022).

## 5 HIF-1α and the mechanism of OS metastasis

In normoxic and hypoxic environments, HIF-1α exhibits distinct pathological mechanisms and actively participates in molecular mechanism regulation of tumor cells through corresponding signaling pathways (Jin Z. et al., 2023b; Ceranski et al., 2023; Li Y. et al., 2023c; Xie et al., 2023). Recent studies have shown that HIF-1α genes-related and signal pathways are significantly associated with OS cells metastasis (Zhao et al., 2019; He G. et al., 2023a). It can induce and sustain distant metastasis of OS cells by modulating the invasive and metastatic potential of tumor cells, promoting the EMT process, enhancing cellular adhesion, increasing anti-apoptotic properties, inducing immune evasion, facilitating tumor angiogenesis, and fostering microenvironmental remodeling (Table 1).

**TABLE 1:** The role of HIF-1α signaling pathway in the metastasis mechanism of OS cells.

Classification	Signal pathway	Mechanism	References
Invasive potential	CXCR4/HIF-1α	Increases OS cell invasiveness	Zhang et al. (2018)
	CCL4/HIF-1α/integrin αvβ3	Induces cell migration	Lan et al. (2017)
	HIF-1α/miR-18b-5p/ PHF2	Increases cell invasion	Wang et al. (2019b)
	HIF-1α/NUSAP1	Enhances cell migration and invasion	Liu et al. (2016)
	GRM4/CBX4/HIF-1α	OS cell proliferation, migration and invasion	Nieto et al. (2016)
	HIF-1α/FOXD2-AS1/p21	Increases cell invasiveness	Wu et al. (2019)
	miR-33b/HIF-1α	OS cell proliferation and migration	Zhou and He (2022)
	HIF-1α/ANGPTL4	Enhances OS cell proliferation and migration	Tan and Zhao (2019)
	HIF-1α/ANGPTL2	Enhances the invasive ability	Sun et al. (2015)
	miR-20b/HIF-1α/VEGF pathway	OS cell invasion and proliferation	Thüring et al. (2023)
	DEC2/HIF-1α	Facilitates OS cells in response to hypoxia; invasion and metastasis	Shen et al. (2023)
EMT process	HIF-1α/BNIP3/LC3B	Mitochondrial autophagy	Ren et al. (2019)
	HIF-1α/VEGF	Enhances migration and invasion ability	Hamidi and Ivaska (2018)
	lncRNA NEAT1/miR-186-5p/HIF-1α	Enhances proliferation, invasion, and EMT	Harjunpää et al. (2019)
	SENP1/HIF-1α	Enhances EMT process	Janiszewska et al. (2020)
	AKT/mTOR/ HIF-1α	Enhances EMT process	Leng et al. (2016)
	p38/MAPK/HIF-1α		
	HIF-1α/Snail/MMP-9	Enhances EMT process	Itoh et al. (2018)
Adhesion ability	MEK/ERK1/2/HIF-1α/ICAM-1	Lung colonization; promotes glycolysis; adhesion	Feng et al. (2016)
Anti-apoptosis	HIF-1α/FoxO1/Mn SOD/catalase/Sesn3	Promotes cell growth and migration while inhibiting apoptosis	Zhang et al. (2023)
Metabolism	HIF-1α/Hsa-circ-0000566/VHLE3	Promotes OS cells glycolysis	Zhang et al. (2017d)
	miR-543/PRMT9/HIF-1α	OS cells glycolysis	Dou et al. (2021)
	MiR-186/ HIF‑1	Glucose uptake, and lactic acid production in OS cells	Yu et al. (2019)
Vascular remodeling	GIT1/ p-ERK/HIF-1α	VEGF release and angiogenesis	Li et al. (2018b)
	WISP-1/FAK/JNK/HIF-1α/VEGF-A	Promotes angiogenesis	Watson et al. (2021)
	CCL5/CCR5/PKC/c-Src/HIF-1α	Promotes angiogenesis	Sukumar et al. (2013)
	LncRNA MALAT1/mTOR/HIF-1α	Promotes angiogenesis	Liao et al. (2020)
	LncRNA TUG1/miR-143-5p/HIF-1α	Promotes angiogenesis	Zhao et al. (2015)

### 5.1 Invasion ability and OS metastasis

**FIGURE 4:**
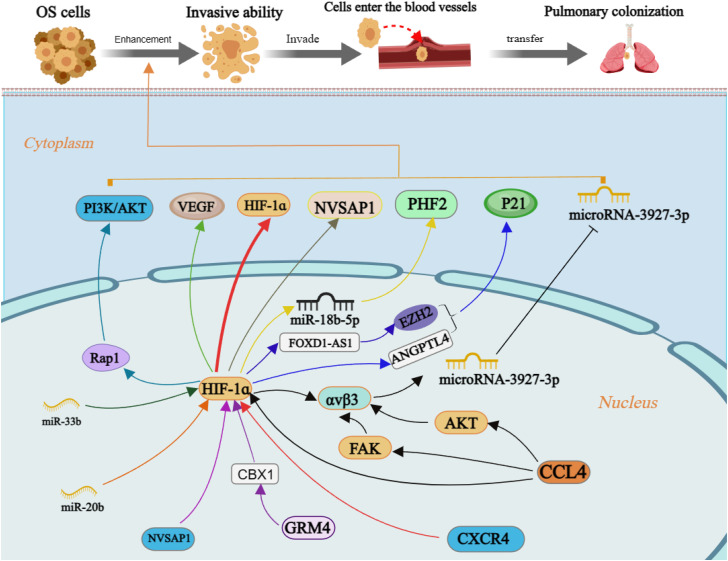
The molecular mechanism of enhancement invasiveness of OS cells by HIF-1ɑ. Annotation: HIF-1α is regulated by many factors and regulates the level of downstream target factors, which can enhance the invasive ability of OS cells and increase the risk of distant metastasis.

Tumor cells possess the capacity for invasion and migration during the early stages of metastasis, enabling them to detach from the primary tumor mass and infiltrate neighboring and distant host tissues (Kushwaha et al., 2019). The initial phase of metastasis can be completed by HIF-1α by increasing the invasive ability of OS cells (Figure 4). It was established that hBMSC-MVs augment the invasiveness of OS cells under hypoxic conditions through activation of the HIF-1α pathway (Lin et al., 2019). An intriguing study unveiled a correlation between HIF-1α and C-X-C chemokine receptor type 4 (CXCR4), a chemokine receptor, in the context of OS metastasis. Hypoxia-induced high-metastatic potential OS cells exhibit heightened invasiveness compared to low-metastatic potential counterparts, with their induction being sensitive to CXCR4 antagonists and HIF-1α inhibitors (Guan et al., 2015).

Moreover, OS clinical stage and lung metastasis are positively linked with Chemokine C-C motif ligand 4 (CCL4) and integrin αvβ3 expression. CCL4 promotes the development of integrin αvβ3 and enhances cell migration potential in OS by activating the HIF-1α, PKB, and focal adhesion kinase (FAK) signaling pathways while suppressing miRNA-3927-3p expression (Tsai et al., 2021). Hypoxia-induced upregulation of miR-18b-5p through HIF-1α transcriptional control inhibits tumor suppressor gene PHD finger protein 2 (PHF2) expression at the post-transcriptional level; its suggested that the miR-18b-5p-PHF2 signal axis is involved in the HIF-1α-mediated metastasis of OS (Luo et al., 2022). The upregulation of both HIF-1α and nucleolar and spindle associated protein 1 (NUSAP1) in OS cells positively correlates with their invasive capacity under hypoxic conditions (Zhang et al., 2021). Although bioinformatics analysis did not show significant differences in glutamate metabotropic receptor 4 (GRM4) expression between OS and normal tissue. However, subsequent cell tests revealed that the interaction between GRM4 and Chromobox 4 had a notable impact on the transcriptional activity of HIF-1α. Overexpressing GRM4 resulted in a substantial reduction in cell proliferation, migration, and invasion ability (Zhang et al., 2020).

The P21 protein is a cell cycle-dependent protein related to the invasive ability of cells (Cao et al., 2020). In OS cells, the interaction between FOXD1-AS1 and ZEST homologous enhancer 2 suppresses the level of p21 protein. Simultaneously, the expression of FOXD1AS1 is regulated by the transcription factor HIF-1α, which plays a crucial role in augmenting the malignant biological characteristics of OS cells (Ren et al., 2019). MiR-33b has been identified as targeting HIF-1α, and its downregulation in OS cells enhances cell proliferation and migration. Overexpression of HIF-1α reverses the inhibitory effect of miR-33b on cell proliferation and migration (Zhou et al., 2017). Suppressing CircRNA-103801 expression can reduce OS cell proliferation, migration, and invasion capacity. Downregulation of miR-338-3p may lead to upregulation of CircRNA-103801 expression in OS cells through primarily involving the circRNA_103801-miR-338-3p-HIF-1/Rap1/PI3K-Akt pathway; unfortunately, it is not clear which subtype of HIF-1 was involved in this study (Li ZQ. et al., 2021c).

Angiotensin like 4 (ANGPTL4) is associated with the vascular remodeling and regeneration (Fernández-Hernando and Suárez, 2020). The expression of ANGPTL4 is upregulated by HIF-1α and thus enhances OS cell proliferation and migration (Z et al., 2018). Quercetin can suppress OS cell migration and invasion in a dose- and time-dependent manner by decreasing HIF-1α, VEGF, matrix metalloproteinase 2 (MMP2) and MMP9 expression (Lan et al., 2017). It has been indicated that the expression of angiopoietin Like 2 (ANGPTL2) is upregulated in HIF-1α-induced OS cells, and ANGPTL2 overexpression increased OS cells’ capacity for invasion (Wang X. et al., 2019b). Meanwhile, miR-20b significantly decreases in OS cells, resulting in overexpression of the target gene HIF-1α and subsequent upregulation of the VEGF pathway. Thereby promoting cell proliferation and invasion (Liu et al., 2016). Consequently, acquisition of invasive capacity in OS cells trigger the initiation of distant metastasis.

### 5.2 EMT and OS cells metastasis

**FIGURE 5:**
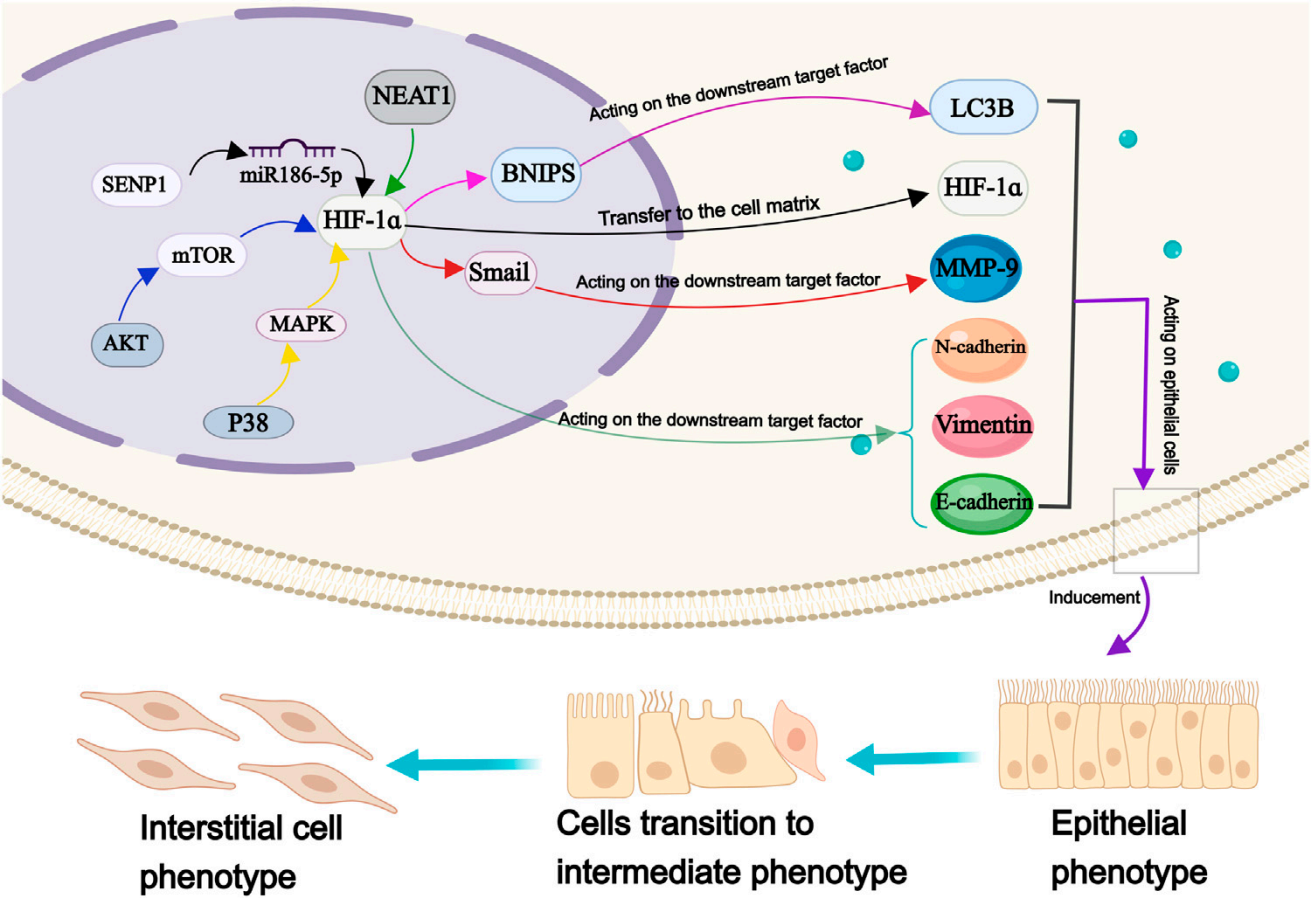
The molecular mechanism of HIF-1ɑ in epithelial mesenchymal transformation of OS cells. Annotation: HIF-1 α is involved in the regulation of many factors, which metastasis to the cell matrix, induce the EMT of OS cells and maintain the transformation process.

EMT is commonly recognized as a critical step in the development of malignant tumors, characterized by the downregulation of epithelial markers and upregulation of mesenchymal proteins. Consequently, EMT results in the loss of epithelial polarity, relaxation of intercellular junctions, and recombination of cytoskeleton proteins, ultimately facilitating tumor invasion and metastasis (Nieto et al., 2016). Remarkably, HIF-1α has been demonstrated implicated in regulating the progression of EMT (Figure 5).

Under hypoxia, the expression of E-cadherin is upregulated, while the expression of Vimentin, N-cadherin, and snail protein is downregulated; all these proteins serve as markers for EMT (Zhang et al., 2020). With the expression of HIF-1α and TWIST family bHLH transcription factor 1 is upregulated, while the expression of e-cadherin is downregulated, leading to altered EMT process in OS cells (Fujiwara et al., 2020). Previous studies have reported that HIF-1α can interact with β-catenin to form HIF-1α/β-catenin complex, promoting tumor cell proliferation and metastasis (Wu et al., 2019). Interestingly, a cellular investigation has revealed a phenomena that defies the foregoing conclusion has been discovered. Their research findings suggested that the regulation of OS cell metastasis involved the mitochondrial autophagy degradation pathway, which was mediated by HIF-1α/BCL2/adenovirus E1B interacting protein 3/light chain 3 beta. The aforementioned mechanism may provide an explanation for the underlying cause of this apparent contradiction (He G. et al., 2023a). Research has shown significant variations in the expression levels of VEGF, E-cadherin, and N-cadherin in OS cells, corresponding to changes in HIF-1α level, thereby leading to a substantial enhancement in their migratory and invasive capabilities. However, *sauchinone* effectively mitigates the detrimental effects of hypoxia-induced EMT on OS cells (Zhou and He, 2022). miR-186-5p functions as a downstream target of nuclear enriched abundant transcript 1 which targets HIF-1α. It inhibits proliferation, invasion, and EMT through miR-186-5p/HIF-1α axis in OS cells (Tan and Zhao, 2019).

In the anoxic microenvironment, both HIF-1α and UMO specific-protease 1 (SENP1) exhibit upregulation in OS cells. Interestingly, inhibition of HIF-1α result in the suppression of SENP1 enhancement. Subsequent inhibition of SENP1 leads to the upregulation of E-cadherin and downregulation of vimentin and N-cadherin, thereby regulating EMT and attenuating the invasive potential of tumor cells (Wang et al., 2018). *Tetrahydrocurcumin (THC)* reduces HIF-1α levels under hypoxic conditions by blocking the Akt/mTOR and p38/MAPK pathways, thus improving the EMT process (Zhang Y. et al., 2017a). Another study found that *Melatonin* inhibits EMT in OS cells through HIF-1α/Snail/MMP-9 signal transduction, thereby inhibiting OS metastasis (Chen et al., 2020). When exposed to hypoxia, silencing of HIF-1α restore the upregulation of E-cadherin expression and downregulates vimentin expression in OS cells, significantly impacting their proliferative and invasive abilities. *Resveratrol* prevented the development of HIF-1α protein without altering HIF-1α mRNA levels (Sun et al., 2015).

The process of EMT assumes a paramount significance in enabling tumor cells to acquire invasive abilities, thus serving as an essential node in initiating and spreading malignant tumors. The cytokine HIF-1α plays a crucial role in regulating the process of EMT. Therefore, focusing on modulating the HIF-1α-regulated EMT process will offer a novel therapeutic approach for treatment of metastatic OS.

### 5.3 Cell adhesion and OS metastasis

Adhesive changes are an important feature of malignant tumors (Liu et al., 2022; Thüring et al., 2023). Due to the lack of cellular adhesion, tumor cells possess the capacity to infiltrate neighboring tissues as individual cells or small clusters, thereby enhancing their potential for distant metastasis (Gkretsi and Stylianopoulos, 2018; Cao et al., 2023). Cell adhesion encompasses cell-cell, cell-extracellular matrix, and cell-protein interactions (Hamidi and Ivaska, 2018). Integrin, cadherin, selectin, and IgCAM are the key regulators involved in cell adhesion (Harjunpää et al., 2019). E-cadherin, integrin, and epithelial cell adhesion molecules are closely related to EMT and tumor cell migration; selectin and IgCAM may serve as important factors enabling tumor cells to evade immune surveillance (Janiszewska et al., 2020). Targeting cell adhesion molecules is a popular approach in antitumor therapy, particularly focusing on inhibiting integrins (Hamidi and Ivaska, 2018). Surprisingly, the mechanism of cellular adhesion has been elucidated in the dissemination of certain malignant tumors (Leng et al., 2016). The discovery offers promising prospects for the management of metastatic malignant tumors.

**FIGURE 6:**
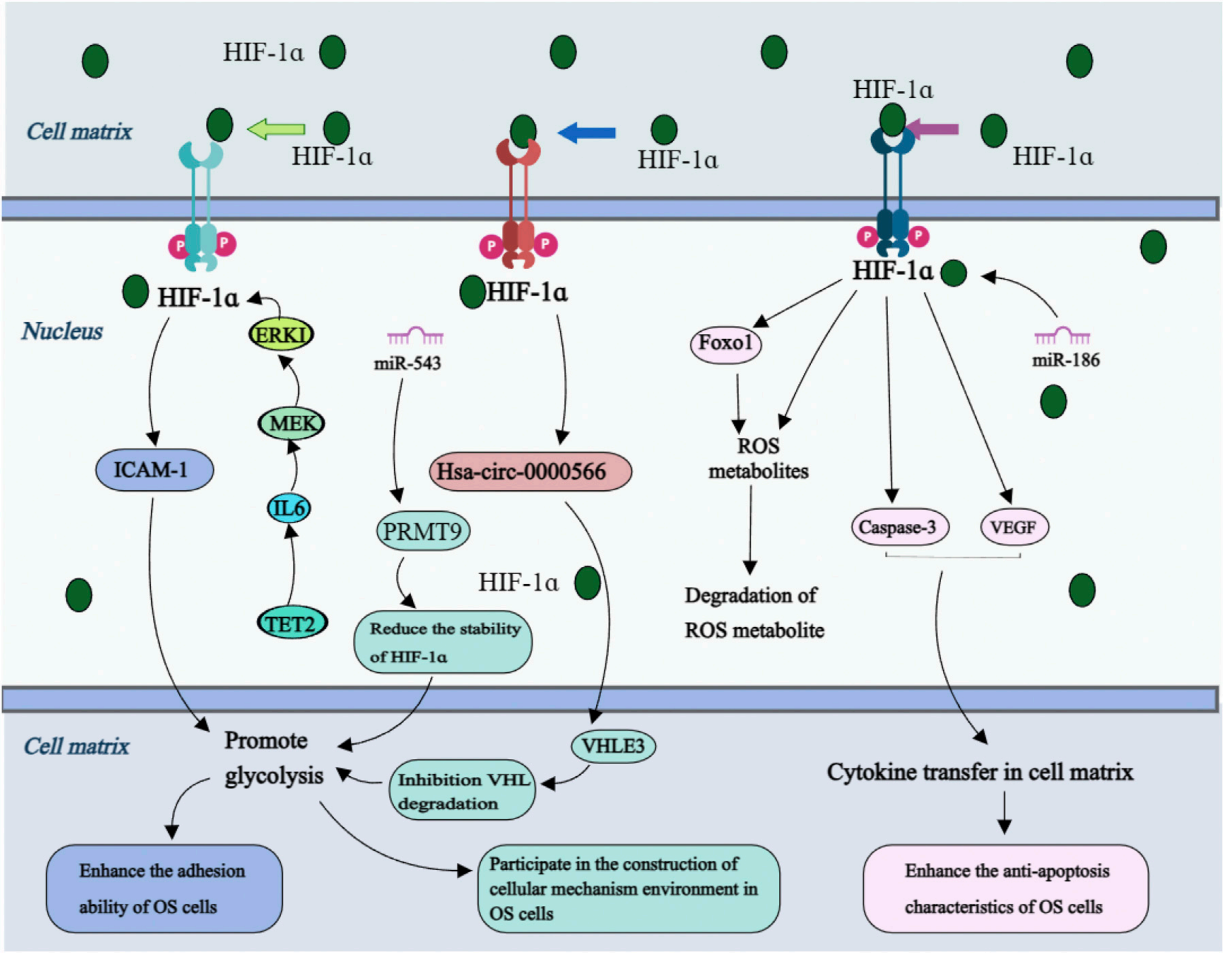
Molecular mechanism of HIF-1ɑ receptor in OS cell adhesion, anti-apoptotic properties and matrix environment construction. Annotation: On the one hand, changes in the level of downstream factors regulated by HIF-1α and changes in the cellular matrix environment such as the degradation of reactive oxygen species enhance the ability of cells to resist apoptosis. On the other hand, the downstream factors regulated by HIF-1α participate in the glycolysis process of OS cells, enhance cell adhesion and participate in the construction of a suitable environment for OS cell growth, guide OS cells to break through local lesions, colonization, and complete the process of metastasis.

In the mechanism of bone tumor metastasis (Figure 6), intercellular adhesion molecule-1 (ICAM-1) expression is connected to HIF-1α, IL-6 enhances ICAM-1 expression through activation of the MEK/ERK1/2/HIF-1α pathway in OS cells, and Tet methylcytosine dioxygenase 2 contributes to demethylation and IL-6 upregulation in tumor cells (Itoh et al., 2018). The alteration in the process of EMT, as indicated by the change in cadherin expression, frequently occurs concurrently with loss of cellular adhesion (Almotiri et al., 2021; Fujisaki and Futaki, 2022). These findings suggest a potential association between OS cell adhesion, EMT process and OS cells invasiveness.

### 5.4 Cell apoptosis and OS metastasis

Apoptosis is one of the main pathways of programmed cell death, which can be triggered by multiple extracellular and intracellular factors (Pihán et al., 2017). Enhanced antiapoptotic properties alter tumor cell characteristics and elevate the risk of distant metastasis events (Figure 6). It has been shown that HIF-1α can directly regulate ForkheadboxClassO1 in OS cells, prevent the accumulation of reactive oxygen species (ROS), and inhibit manganese-dependent superoxide dismutase, catalase, and Sesn3. Hypoxia-induced ROS formation and apoptosis in OS cells are associated with interference in the cytochrome P450 enzyme system. The HIF-1α inhibitor 2-mercaptoethanol and ROS inducer arsenic oxide inhibit OS cell proliferation and migration while promoting apoptosis (Sun et al., 2019). *In vitro* experiments have confirmed that HIF-1α downregulates caspase-3 expression, promotes OS cell growth, and inhibits apoptosis processes (Lv et al., 2016).

The transcription factor DEC2 gene is associated with a shortened sleep rhythm and plays a role in regulating tumor pathology as a transcriptional suppressor (He et al., 2009). It has been demonstrated that DEC2 expression is significantly correlated with HIF-1α levels. In OS cell lines, knockdown of DEC2 reduces HIF-1α accumulation and compromise the ability of HIF-1α to activate its target genes in response to hypoxia. Increased expression of DEC2 causes OS cells to accumulate HIF-1α more quickly, which aids their adaptation to anoxic environments (Hu et al., 2015). Additionally, the death mechanism of OS cells is supplemented by HIF-1α-induced autophagy. As a result of prolyl hydroxylase being inhibited by ROS and Zn, autophagy is demonstrated to be triggered in OS cells via the connection between HIF-1α and the autophagy-zinc-ROS autophagy cycle axis (He et al., 2020).

Radiation therapy is a treatment modality employed for the management of malignant tumor cells. The administration of HIF-1α siRNA treatment significantly diminishes the hypoxia-induced antiradiation potential of OS cells (Jin et al., 2015). Under hypoxic conditions, HIF-1αoverexpression expedites ROS clearance through autophagy induction in OS cells, thereby conferring radiation resistance to these cells (Feng et al., 2016). Targeting the HIF-1α pathway may enhance radiation resistance induced by hypoxia (von et al., 2021). Anti-apoptotic properties increase the potential of OS distant metastasis. The HIF-1α signaling pathway plays an important role in regulating OS cell apoptosis. Regulating related signal factors in order to promote tumor cell apoptosis allows for new treatments for metastatic OS.

### 5.5 Establishment of microenvironment and OS metastasis

Malignant tumors can proliferate rapidly, and the increasing oxygen consumption of tumor cells perpetuates their hypoxic environment. Within this hypoxic milieu, tumor cells obtain energy through glycolysis, i.e., the Warburg effect (Liberti and Locasale, 2016). Cell metabolism undergoes different stages during distant malignant tumor cell metastasis, providing energy and essential metabolites for the continuous growth and proliferation of cancer cells (Phan et al., 2014). The metabolic activity of tumor cells fosters an advantageous environment for the biological activities of malignant cells (Zhang et al., 2023). Simultaneously, through a dynamic multi-step metastasis cascade, the communication between the structural and cellular components within the tumor microenvironment (TME) induces cancer cells to disseminate from their primary site to distant areas (Neophytou et al., 2021). Following colonization in distant organs, metastatic cells interact with the TME, which involves angiogenesis and other processes, and the metabolic process is reprogrammed to achieve metastatic tumor cell growth (De Palma et al., 2017; Zanconato et al., 2019).

In the process of OS metastasis (Figure 6), Hsa-circ-0000566 is regulated by HIF-1α, and both directly binds to VHLE3 ubiquitin ligase protein to inhibit VHL-mediated ubiquitin degradation, thereby promoting their pivotal role in glycolysis of OS cells (Shen et al., 2023). Mechanistically, protein arginine methyltransferase 9 (PrMT9) exerts control over HIF-1α through a distinct mechanism. miR-543 suppresses oxidative phosphorylation, which is promoted by PrMT9, and deletion of miR-543 enhances the PrMT9-induced destabilization of HIF-1α, leading to inhibition of glycolysis in OS cells (Zhang H. et al., 2017b). It has been shown that miR-186 regulates the expression of HIF-1α by targeting pituitary tumor transforming gene 1, thus facilitating glucose uptake and promoting lactic acid generation (Xiao et al., 2018).

The remodeling of the cellular microenvironment, as mentioned in the mechanism of malignant tumor metastasis, not only facilitates the acquisition of invasive ability by tumor cells but also plays a crucial role in sustaining metastasis and facilitating host organ colonization. This substantiates that the TME induced by HIF-1α is related to maintaining the stability of all OS cell metastasis stages, HIF-1α regulation is an important way to inhibit distant OS metastasis.

### 5.6 Vascular remodeling and OS metastasis

**FIGURE 7:**
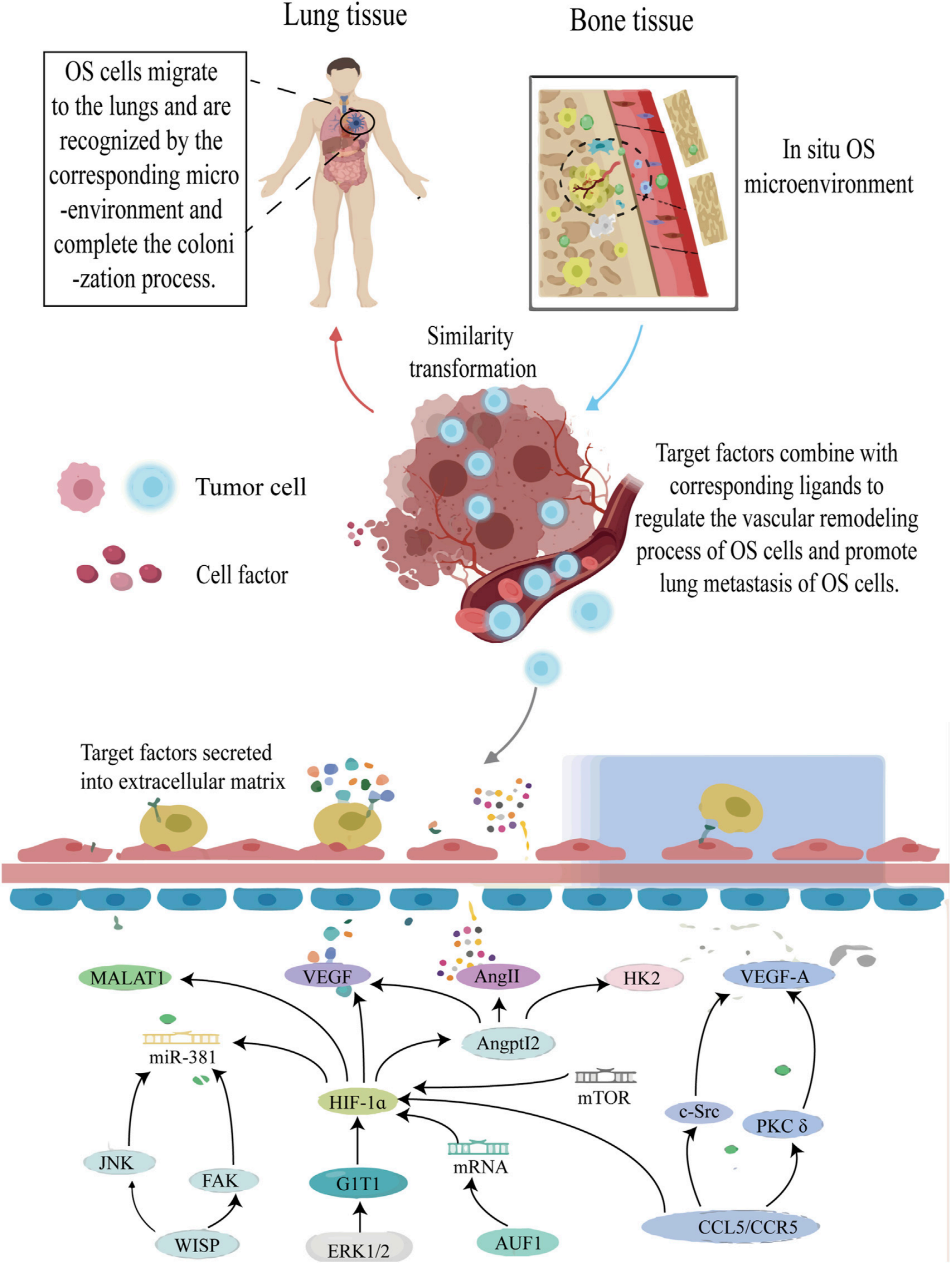
The roles of HIF-1ɑ in vascular remodeling of OS cells. Annotation: HIF-1α-induced signal pathway regulates the vascular remodeling process of OS tissue and improves the anoxic environment of OS cells. At the same time, the vascular remodeling process will undoubtedly increase the risk of metastasis of OS cells.

Vascular remodeling is an important factor that impacts tumor cell growth and metastasis by facilitating oxygen and nutrient supply to the cancer cells, as well as supporting their infiltration and extravasation. Tumor angiogenesis is triggered by environmental stress, with hypoxia being the most significant contributor. Environmental stress leads to the imbalance of promoting/anti-angiogenesis, which leads to increased expression of angiogenic factors (Corre et al., 2020). Although OS is a highly vascularized bone tumor, the mechanism of neovascularization in OS remains unknown. Studies have shown that HIF-1α plays an important role in vascular remodeling of OS (Figure 7).

The research findings confirmed that in addition to increasing OS cell invasion capacity, HIF-1α mediates ANGPTL2 expression and promotes VEGF, Angie, and Hexokinase 2 synthesis *in vitro* and *in vivo*. These factors are related to vascular remodeling in OS cells (Wang X. et al., 2019b). ERK1/2 is a key player in G-protein-coupled receptor interacting protein 1 (GIT1)-mediated VEGF secretion and angiogenesis, while HIF-1α acts as the primary transcription factor controlling VEGF production. Under hypoxia, HIF-1α expression can be observed; however, its deletion by GIT1 drastically reduces its expression by preventing ERK1/2 activation. In such circumstances, although VEGF levels also decrease, the downregulation of HIF-1α expression occurs. Therefore, the researchers hypothesized that the ERK1/2/HIF-1α pathway could be utilized to elucidate this mechanism (Zhang et al., 2018).

The transcriptional activities of HIF-1α and activator protein-1 (AP-1) are regulated by α-CaMKII. By encouraging the binding of VEGF transcript with HIF-1α and AP-1, VEGF expression is increased, which aids in the neovascularization of OS (Daft et al., 2015). HIF-1α, FAK, and Jun N-terminal kinase signal pathways are activated upon stimulation of WNT1-induced signal pathway protein-1 (WISP-1). The interaction between integrin and WISP-1 contributes to the control of vascular regulation, increase VEGA production in human OS cells and stimulate angiogenesis and migration in human endothelial progenitor cells (Tsai et al., 2017). Additionally, HIF-1α gene suppression through siRNA can drastically lower VEGF protein levels and VEGF mRNA expression at the transcriptional level, effectively suppressing angiogenesis (Zhang XD. et al., 2017c). In previous studies, ribonucleic acid binding protein AUF1 directly binds and stabilizes the positive regulatory factor HIF-1α and VEGF-A gene, and positively regulates the expression of both genes, which enhances the angiogenic ability of OS cells *in vitro* and *in vivo*. This effect is caused by inoculating AUF1 protein into the sequence of VEGF-A and HIF-1α (Al-Khalaf and Aboussekhra, 2019).

Interestingly, the involvement of THC in the HIF-1α-mediated EMT process is not only evident but also demonstrates a partial inhibitory effect on OS vascular remodeling (Zhang Y. et al., 2017a). It has been indicated that the HIF-1α-mediated PKC/c-Src/HIF-1α signaling pathway is activated by Cmurc chemokine ligand 5/C-C chemokine receptor 5, leading to an increase in tumor angiogenesis dependent on VEGF in the OS microenvironment (Wang et al., 2015). In a fascinating study, the involvement of HIF-1α in the regulation of the rapamycin mTOR/HIF-1α/metastasis-related lung adenocarcinoma transcript 1 signaling axis has been confirmed, highlighting its significance in vascular remodeling in OS (Zhang ZC. et al., 2017d). Subsequent research revealed increased expression of HIF-1α, monocyte chemoattractant protein-1 (MCP1), MCP2, MCP3, Lmur6, and VEGF in OS cells associated with vascular remodeling along the osteosarcoma’s metastatic pathway (Dou et al., 2021). Furthermore, the process of vascular remodeling in OS is regulated by taurine upregulation of gene 1 (TUG1), which is owing to a competitive binding mechanism, TUG1 competitively protected HIF-1α from miR-143-5p, and is expected to be the prognostic indicator and therapeutic target of OS (Yu et al., 2019).

Another mechanism of vascular remodeling in tumor cell metastasis is tumor cell infiltration and extravasation. During a specific stage of capillary endothelial cell remodeling, cancer cells successfully exudate and form new capillary rings, which is characteristic of invasive malignant tumor metastasis (Giordo et al., 2022; Karreman et al., 2023). Unfortunately, limited knowledge exists regarding this pathway in terms of OS metastasis.

Vascular remodeling plays a crucial role in the adaptive development of tumor cells, contributing to the establishment of a metastatic microenvironment for solid tumors (Li X. et al., 2018b). Inhibition of angiogenesis in solid tumors can induce apoptosis in OS cells; thus, preventing vascular remodeling represents a promising strategy to enhance the prognosis of metastatic OS.

### 5.7 Immune escape and OS metastasis

As previously mentioned, the majority of solid tumor cells are in a hypoxic state due to their malignant characteristics. The hypoxia of microenvironment is associated with immune escape and immune cell apoptosis (Vito et al., 2020). During glycolysis, lactic acid is produced in the extracellular matrix, and increased lactic acid levels can inhibit T cell function and enhance tumor cell anti-immune response (DePeaux and Delgoffe, 2021; Watson et al., 2021). Hypoxic metabolism induces an immunosuppressive TME that augments T cell hyperresponsiveness. Specifically, HIF-1α specifically inhibits immune cell function in the TME (Multhoff and Vaupel, 2020). This enables the tumor to escape immune-mediated killing, thus reducing the efficacy of many immunotherapy methods (Vito et al., 2020). Conversely, HIF-1α inhibition can enhance T cell anti-tumor activity and inhibit glycolysis, and reducing HIF-1α levels in the TME can increase memory T cell production and improve anti-tumor function (Sukumar et al., 2013). The aforementioned phenomenon accelerates the metastasis of tumor cells and reduces their consumption involved in the metastatic process (Liao et al., 2020). Unfortunately, the mechanism of immune evasion in HIF-1α-mediated OS transmission has not been reported and requires further investigation.

## 6 Therapeutic prospect of targeting HIF-1α in the treatment of metastatic OS

Metastatic events are crucial factors influencing the unfavorable prognosis in OS, and effective management of metastatic risk holds particular significance for prognosis. It is worth considering that when OS undergoes metastasis, treatment should not solely focus on the primary tumor but also aim to inhibit its invasion and metastasis. This underscores the necessity to comprehend the shared characteristics of primary tumors, tumor metastasis, and secondary sites of metastasis (Du et al., 2023). Research has showed that HIF-1α participates in nearly all processes involved in OS metastasis and serves as a pivotal target within the respective molecular mechanisms. Modulating HIF-1α levels may potentially achieve inhibition of OS metastasis. Interestingly, COX regression analysis confirmed again that HIF-1α expression is a prognostic indicator for the survival of patients with OS. It promotes OS cell invasion by increasing the production of VEGF, which is a promising therapeutic target for metastatic OS prognosis (Zhao et al., 2015).

### 6.1 HIF-1α and gene therapy

In targeted gene therapy, HDACIs are traditionally considered as effective tumor inhibitors as their effect via loosening tightly-wound chromatin to inhibit a variety of tumor suppressor genes. bSAHA, an FDA-approved I/IIb/IV HDACI drug, effectively inhibits the nuclear translocation of HIF-1α by directly acetylating Hsp90 and significantly reduces its transcriptional activity (Zhang C. et al., 2017e). However, it is important to investigate whether SAHA can also inhibit OS metastasis. While current research is limited to autophagy, paclitaxel has demonstrated its ability to target HIF-1α and reduce its activity in OS cells (Guo et al., 2015). Furthermore, px-478 has been reported to modulate the transcriptional level of HIF-1α (Koh et al., 2008). Similarly, the compound YC-1 possesses the capacity to inhibit HIF-1α’s transcriptional activity and effectively impede protein accumulation (Hu et al., 2020). A potent inhibitor of HIF-1, echinomycin is a small-molecule antibiotic derived from the bacterium *Streptomyces*. It competitively hinders the function of HIF-1α by binding to the HRE region. Unfortunately, subsequent research revealed its hazardous nature (Infantino et al., 2021; Zhao et al., 2024). Currently, the creation of echinococcin liposomes has the potential to improve drug accessibility and safety while reducing tumor growth and metastasis (Bailey et al., 2020). The aforementioned statement exemplifies the feasibility of developing HIF-1α-targeted inhibitors that exhibit both advantageous and safe effects.

### 6.2 HIF-1α and vascular remodeling

Inhibiting tumor vascular remodeling is a crucial approach employed to suppress OS metastasis. VEGF serves as an important target of OS in anti-vascular remodeling, which affects almost every step of metastasis. The therapeutic options for OS anti-angiogenesis encompass VEGF monoclonal antibody (bevacizumab), tyrosine kinase inhibitors (sorafenib, apatinib, pazopanil and rigofinib), and recombinant human endostatin (Endostar) (Liu Y. et al., 2021b). Interestingly, bevacizumab not only inhibits vascular remodeling but also participates in immune mechanism regulation (Park et al., 2023). Recent studies have shown that proprietary Chinese medicines can effectively inhibit vascular remodeling in OS. For instance, the synthesis of vancomycin derivative T-methyl chloride can stabilize HIF-1α protein and activate its transcriptional activity, thereby inducing gene expression of downstream targets such as VEGF and GLUT-1 (Magae et al., 2019). Moreover, curcumin exerts multiple inhibitory effects on OS metastatic cells including cell adhesion and vascular remodeling (Zahedipour et al., 2021). Therefore, anti-angiogenesis may simultaneously target multiple molecular mechanisms involved in the process of OS metastasis, making it the most promising therapeutic approach for inhibiting OS metastasis.

### 6.3 HIF-1α and immunotherapy

Immunotherapy is a novel approach employed in the treatment of OS in recent years, and yielding promising outcomes (Tian et al., 2023). OS cells are enveloped by a complex immune microenvironment wherein HIF-1α plays a crucial role in regulating immune factors such as interleukins, transforming growth factor, VEGF, and other elements that significantly contribute to OS metastasis. In the OS cell model, immunotherapy has been observed to augment the efficacy of chemotherapeutic drugs to some extent (Wang H. et al., 2023a; Zhong et al., 2023). Therefore, modulation of the cellular immune microenvironment assumes paramount importance in the management of metastatic OS (Huang et al., 2022; Kuo et al., 2023).

It has been reported that T cells are capable of expressing cytotoxic T lymphocyte-associated antigen-4 (CTLA-4) and programmed cell death protein 1 (PD-1), which are considered as the primary targets of immune checkpoint inhibitors for tumor cells (Conforti et al., 2018). The expression of CTLA-4 exhibits a positive correlation with the prognosis of OS, and the expression level of CTLA-4 is related to the signal pathway of PD-1 activation. Administration of CTLA-4 blockers and antagonists has the potential to restore anti-tumor immunity by activating B7 and CD28 signal transduction pathways (Tian et al., 2023). PD-1 is the key nodes of immune escape of tumor cells, and the expression of PD-1 and its receptor programmed death ligand-1 (PD-L1) is negatively associated with OS prognosis (Zheng et al., 2015; Yang W. et al., 2021c). Treatment with PD-1 inhibitors in OS mice model results in a significant reduction in the probability of lung metastasis (Zheng et al., 2018). Although the role of HIF-1α on PD-1/PD-L1 in OS cells remains unconfirmed, inhibiting the expression of HIF-1α can effectively reduce the expression level of PD-1 in glioma and prostate cancer cells (Ding et al., 2021; Shen et al., 2022). Decreased expression of HIF-1α and PD-L1 promotes the infiltration of CD8 T cells, and increases the levels of TNF-α, IFN-γ, thereby significantly enhancing the efficacy of anti-PD-1 therapy in gastric cancer cells (Wang Z. et al., 2023b). Significantly, PD-L1 has been proved to be a downstream target of HIF-1α in hepatocellular carcinoma (Yao et al., 2023). Interestingly, the HIF-1α inhibitor echinomycin not only augments the therapeutic efficacy of anti-CTLA-4 therapy in immunotherapy, but also enhances the immune tolerance function of PD-1/PD-L1 checkpoints in normal tissues. This dual effects serves to eliminate the immune escape mechanism within the tumor microenvironment, thereby facilitating a safer and more effective approach to immunotherapy (Bailey et al., 2022). Thus, the immune escape mechanism mediated by HIF-1α may be involved in the metastatic process of OS. More researches are demanded to investigate the potential role of HiF-1a in the immunotherapy of metastatic OS.

### 6.4 HIF-1α and ROS level regulation

The level of ROS and the activation of HIF-1α exhibit a significant correlation. The primary focus of research on the regulatory mechanism of HIF-1α lies in elucidating the connection between the HIF-1α pathway and ROS, particularly in terms of modulating environmental ROS levels to maintain optimal HIF-1α expression. N-acetylcysteine is an inhibitor of ROS that can inhibit the apoptosis of diosgenin-related p38/MAPK signal transduction. This indicates that ROS plays an important role in diosgenin-induced apoptosis (Zheng et al., 2023). In addition, the class I HDAC inhibitor MS-275 enhances the vulnerability of ROS in sarcoma cells. Acetylhistological analysis showed that MS-275 promotes rapid acetylation of lysine RNA binding proteins and blocked the binding and translation activation of NFE2L2 and the corresponding mRNA target HIF-1α to stress granule nucleator G3BP1, thus promoting translation control of key cytoprotective factors and inhibiting the metastatic activity of OS cells (El-Naggar et al., 2019). Increased ROS levels not only promoted the iron death process of OS cells but were also significantly correlated with cisplatin resistance (He P. et al., 2023b). D-arginine is the inert metabolic enantiomer of L-arginine, which can produce nitric oxide, enhance oxygen activity in the cell matrix, downregulate HIF-1α, reduce tumor hypoxia, and increase the sensitivity of OS to radiotherapy, as well as enhance the effect of tumor ablation and effectively prevent lung metastasis (Du et al., 2021).

Gene therapy, immunotherapy and optimization of the cellular microenvironment (including regulation of angiogenesis and ROS concentration) can effectively regulate the level of HIF-1α, which is very important to improve the prognosis of metastatic OS. It is conceivable to develop therapeutic targets or interventions that are both safe and efficient, albeit the research in this domain is still in its nascent stages. This underscores the imperative for conducting a substantial amount of research to establish efficacious and targeted therapy options for successful treatment of metastatic OS.

## 7 Conclusion

The development and subsequent metastasis not solely determined by genetic alterations within the tumor cells, but also by the adaptive advantages conferred by these mutations in the particular environment (Tarin, 2013). Anoxic microenvironment can promote tumor cells invasion and facilitate the formation of a “pre-metastatic niche” resembling “soil” in distant organs, which serves as a colonization site for circulating tumor cells and leads to the occurrence of metastatic lesions (Kakkad et al., 2019; Hussain et al., 2020). The involvement of HIF-1α in facilitating the rapid adaptation of tumor cells to the anoxic microenvironment and thus plays crucial roles in OS metastasis. During these processes, HIF-1α is engaged in activating several important signaling pathways, including FAK, AKT, PI3K-Akt, VEGF, ERK1/2. These findings of the current study suggest that HIF-1α orchestrates the process of OS metastasis through a highly intricate network, encompassing the initiation, maintenance, and distant colonization of metastatic lesions. For better prediction and treatment for OS metastasis, further investigation into the link between HIF-1α and OS metastasis is imperative.

Targeting HIF-1α as a strategy to reverse the metastatic process in OS demonstrates significant potential; however, current challenges still persist. Recent studies indicate that HIF-1α levels can be modulated through appropriate interventions and the utilization of specific pharmaceutical agents. Therefore, the development of HIF-1α inhibitors is expected to offer more promising therapeutic options for patients with metastatic OS in the future. However, the current understanding of HIF-1α in the tumor microenvironment and its corresponding small-molecule inhibitors is still in the developmental phase, yet to progress to the clinical trial stage. In order to facilitate the clinical application of HIF-1α, it is imperative to determine its efficacy, bioavailability, and potential adverse effects in patients with metastatic OS.

In a word, it can be concluded that HIF-1α promotes the initiation and development of OS metastasis though facilitating cell invasion, vascular remodeling, EMT, and immune escape. Targeting HIF-1α has the potential to decelerate the progression of OS metastasis. Hence, it is essential to elucidate of the precise mechanism underlying HIF-1α in OS metastasis. Moreover, further investigation of associated molecular mechanisms may facilitate the development of efficient and safe small-molecule inhibitors for HIF-1α in treatment of patients of metastatic OS.

## Abbreviations

OS, Osteosarcoma; HIF-1α, Hypoxia-inducible factor-1α; PHD, Proly Hydroxylase Domain; VHL, VonHippel-Lindaup; HRE, Hypoxia response element; PI3K, Phosphoinositide 3-Kinase; PKB/AKT, Protein Kinase B; ERK 1/2, Extracellular regulated protein kinases 1/2; EMT, Epithelial-mesenchymal transformation; CCL4, Chemokine C motif ligand 4; FAK, Focal adhesion kinase; GRM4, Glutamate metabotropic receptor 4; CXCR4, C-X-C chemokine receptor type-4; PHF 2, PHD Finger Protein 2; ANGPTL 4, Angiotensin like 4; MMP 2, Matrix metalloproteinase-2; VEGF, Vascular endothelial growth factor; SENP 1, SUMO-specificprotease1; TWIST 1, TWIST family bHLH transcription factor 1; ROS, Reactive oxygen species; TME, Tumor microenvironment; PRMT9, Promotes protein arginine methyltransferases 9; GIT1, G-protein-coupled receptor interacting protein 1; MCP 1, Monocyte chemoattractant protein-1; TUG 1, Taurine upregulated gene 1; ICAM-1, Intercellular adhesion molecule-1; PD-1, Programmed cell death protein 1; CTLA-4, Cytotoxic T lymphocyte-associated antigen-4; PD-L1, Programmed death ligand-1.
